# Generalized Cutaneous Lichen Amyloidosis in a Patient with an Ultra-Rare RET Y806C Variant Associated with MEN2A: A Case Report and Literature Review

**DOI:** 10.3390/jcm15103572

**Published:** 2026-05-07

**Authors:** Nina Łabędź, Anna Wiśniewska-Szymańska, Katarzyna Korecka, Ewelina Szczepanek-Parulska, Bartłomiej Budny, Małgorzata Janicka-Jedyńska, Monika Bowszyc-Dmochowska, Aleksandra Dańczak-Pazdrowska, Adriana Polańska

**Affiliations:** 1 Department of Dermatology, Poznan University of Medical Sciences, 61-701 Poznan, Poland; 2 Doctoral School, Poznan University of Medical Sciences, 61-701 Poznan, Poland; 3 Department of Dermatology and Venereology, Poznan University of Medical Sciences, 61-701 Poznan, Poland; 4 Department of Endocrinology, Metabolism and Internal Medicine, Poznan University of Medical Sciences, 61-701 Poznan, Poland; 5 Department of Clinical Pathology, Poznan University of Medical Sciences, 61-701 Poznan, Poland; 6 Cutaneous Histopathology and Immunopathology Section, Department of Dermatology, Poznan University of Medical Sciences, 61-701 Poznan, Poland

**Keywords:** MEN2A, cutaneous lichen amyloidosis, CLA, Y806C, medullary thyroid carcinoma

## Abstract

Cutaneous lichen amyloidosis (CLA) is a rare dermatological condition characterized by amyloid deposition in the skin, presenting as pruritic, hyperkeratotic papules. Although most cases are sporadic, CLA has been associated with multiple endocrine neoplasia type 2A (MEN2A), a hereditary syndrome caused by germline alterations in the RET proto-oncogene. In MEN2A, CLA is typically localized to the interscapular region and linked to RET codon 634 variants, whereas generalized forms are rare. We report a male patient with MEN2A and a generalized form of CLA that preceded the diagnosis of primary hyperparathyroidism (PHPT) and medullary thyroid carcinoma (MTC). Genetic testing using Sanger sequencing identified an ultra-rare heterozygous RET variant, p.Y806C, in exon 14, currently classified as a variant of uncertain significance (VUS). This variant has not been previously described in association with MEN2A. This case may contribute to understanding genotype–phenotype correlations in MEN2A and suggests that atypical or generalized CLA may be an early clinical clue warranting consideration of RET genetic testing.

## 1. Introduction

Cutaneous lichen amyloidosis (CLA) is the most common form of primary localized cutaneous amyloidosis, characterized by amyloid deposition in the skin and presenting as pruritic, hyperkeratotic papules. Lesions are typically localized, most often involving the extensor surfaces of the lower limbs. The pathogenesis is multifactorial and includes chronic mechanical irritation, environmental factors, and genetic predisposition [1].

A rare form of CLA is associated with multiple endocrine neoplasia type 2A (MEN2A), a hereditary syndrome caused by germline variants in the RET proto-oncogene. MEN2A is characterized by medullary thyroid carcinoma (MTC), with possible co-occurrence of pheochromocytoma and primary hyperparathyroidism (PHPT). CLA in MEN2A is classically localized to the interscapular region and most frequently linked to RET codon 634 mutations [2]. RET variants in MEN2A are most commonly found in exons 10 and 11, particularly at codon 634, whereas less frequent mutations involve other exons, including exon 14.

Here, we present a rare case of MEN2A associated with a generalized form of CLA and PHPT in a patient carrying an ultra-rare RET variant at codon 806 (exon 14, c.2417A>G), currently classified as a variant of uncertain significance (VUS). This case may contribute to the understanding of genotype–phenotype correlations in MEN2A and suggests that the clinical spectrum of CLA associated with RET variants may be broader than previously recognized.

## 2. Case Report

### 2.1. Patient History and Clinical Findings

**Figure 1:**
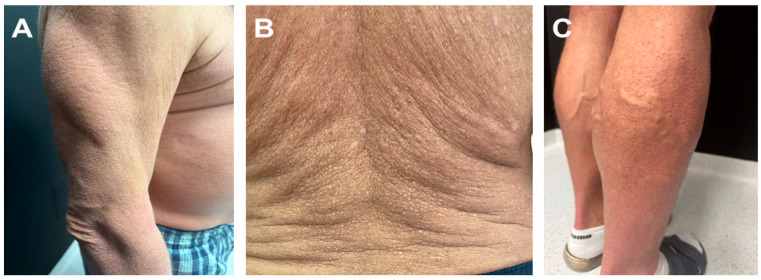
(**A**–**C**) Clinical presentation of MEN2A-associated CLA in our patient. Generalized dry, scaly, hyperkeratotic papular lesions involving the trunk and upper and lower limbs.

A 56-year-old Caucasian male presented to our department with pruritic, hyperkeratotic papules, first noted on both lower extremities at the age of 40. Over the following years, the disease gradually progressed. By 2017, the lesions had become intensely pruritic and progressively increased in both number and distribution, evolving into a generalized pattern. Initially confined to the lower extremities, they later extended to involve the trunk and upper limbs, with increasing hyperkeratosis and coalescence of papules (Figure 1A–C). A skin biopsy performed at that time was interpreted as lichen simplex chronicus.

**Figure 2:**
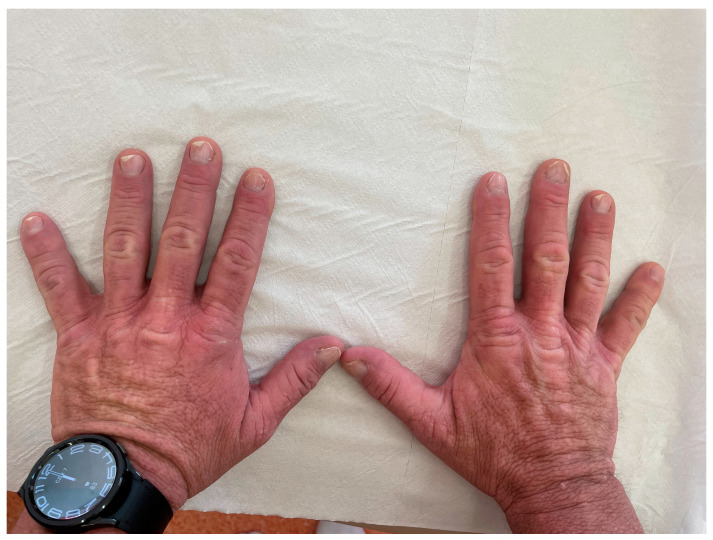
Nail abnormalities in a patient with MEN2A-associated CLA, including onycholysis and onychorrhexis.

Additionally, the patient presented with nail dystrophy. On clinical examination, onycholysis and longitudinal ridging of the nail plates (onychorrhexis) were observed (Figure 2).

**Figure 3:**
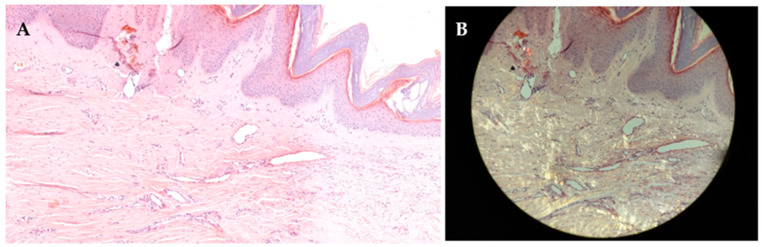
(**A**) Histopathological image of cutaneous lichen amyloidosis (Congo red stain), total magnification ×100. (**B**) Apple-green birefringence under polarized light, total magnification ×200.

Due to persistent symptoms and disease progression, a repeat skin biopsy was performed in August 2022. Congo red staining demonstrated orange-red amyloid deposits in the papillary dermis, with apple-green birefringence under polarized light, confirming the diagnosis of CLA (Figure 3A,B).

**Figure 4:**
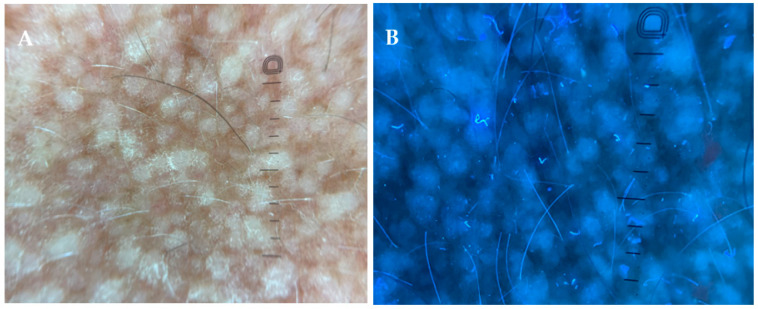
(**A**) Dermoscopic image of cutaneous lichen amyloidosis on the arm, showing a central white to brown hub with surrounding brownish pigmentation (DermLite DL5, polarized light, magnification ×10). (**B**) Ultraviolet-induced fluorescence dermoscopy of a lesion on the arm demonstrating white structureless areas corresponding to amyloid deposition (DermLite DL5, magnification ×10).

Dermoscopy revealed a central white-to-brown hub with surrounding brownish pigmentation. Ultraviolet-induced fluorescence dermoscopy demonstrated white, structureless areas that potentially correspond to amyloid deposition (Figure 4A,B).

Initial management of CLA in our patient included topical corticosteroids, calcineurin inhibitors, keratolytic agents, and emollients, with limited efficacy. Narrowband UVB phototherapy was subsequently initiated, without significant improvement. Systemic therapy with oral acitretin was then introduced, also with minimal clinical response. Overall, the disease remained refractory to treatment.

### 2.2. Physical Examination and Diagnostic Assessment

In June 2020, laboratory investigations were performed due to bone and muscle pain, revealing hypercalcemia (total calcium 11.29 mg/dL; reference range 8.80–10.20 mg/dL), elevated ionized calcium (6.06 mg/dL; reference range 4.20–5.20 mg/dL), and increased parathyroid hormone (PTH) levels (110 pg/mL; reference range 15–65 pg/mL), along with normal phosphate levels. Based on these findings, PHPT was diagnosed.

Despite repeated localization attempts, including technetium-99m sestamibi scintigraphy and cervical ultrasound, no clear parathyroid adenoma was identified. Computed tomography also failed to reveal definitive pathological findings. Due to persistent biochemical abnormalities and clinical symptoms, further evaluation with ^18F-fluorocholine PET/CT was performed, which demonstrated focal tracer uptake inferior to the lower pole of the left thyroid lobe, suggestive of a parathyroid lesion.

Parathyroidectomy was subsequently performed in December 2022, with intraoperative parathyroid hormone monitoring confirming an appropriate decrease in PTH levels (to 21.5 pg/mL post-excision). Postoperative calcium levels decreased accordingly. Histopathological examination confirmed a parathyroid adenoma.

During the same surgical procedure, intraoperative exploration of the thyroid gland revealed a small lesion. Histopathological evaluation demonstrated an incidental microfocus of MTC measuring 2.5 mm. The patient was subsequently referred for definitive surgical management.

In April 2023, the patient underwent total thyroidectomy with central lymph node dissection. Final histopathological examination confirmed a 3 mm MTC. Immunohistochemistry showed positivity for calcitonin and CEA, with negative staining for CK19 and thyroglobulin. No lymphovascular or perineural invasion was identified. No lymph node metastases were found following central lymph node dissection (pT1a pN0), and subsequent lateral lymphadenectomy confirmed the absence of nodal involvement. Postoperative calcitonin levels were within the normal range.

No biochemical or imaging evidence of pheochromocytoma was identified during the diagnostic work-up. Given the uncertain genotype–phenotype correlation, ongoing surveillance in accordance with MEN2A clinical guidelines is recommended.

### 2.3. Genetic Testing

**Figure 5:**
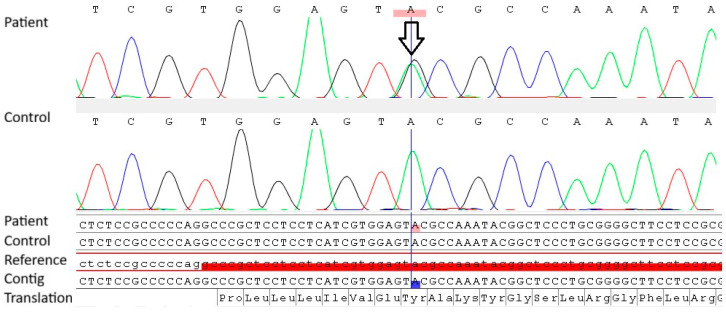
Sanger sequencing chromatogram showing a heterozygous variant in the patient (arrow) and the corresponding reference sequence.

Genetic testing was performed on peripheral blood lymphocytes to identify a germline RET variant, in line with the standard diagnostic approach for MEN2A. Sanger sequencing identified a rare heterozygous variant at codon 806 (exon 14) of the RET proto-oncogene (NM_020975.6:c.2417A>G; p.Tyr806Cys; GRCh38(NC_000010.11): g.43119555A>G; rs377767419), as shown in Figure 5.

Testing was repeated using an independent sample, confirming the presence of the variant. The variant has been submitted to ClinVar by multiple groups and is currently classified as a VUS. Variant interpretation was performed in accordance with the ACMG/AMP guidelines [3]. Based on available evidence, the variant meets several supporting and moderate criteria (PM1, PM6, PP2, PP3, and PP4), while benign criteria (BA1, BS1, BS2) were not fulfilled. Given its ultra-rare frequency in population databases such as gnomAD and reports in affected individuals, criterion PS4 may be considered at a supporting level; however, this remains uncertain due to the limited number of reported cases. In silico prediction tools (including FATHMM, CADD, MutationTaster, REVEL, MutationAssessor, PolyPhen-2, and DANN) consistently suggest a potentially deleterious effect on protein function. Overall, while these findings support a possible pathogenic role, the variant remains classified as a VUS in the absence of functional validation and broader segregation data.

**Figure 6:**
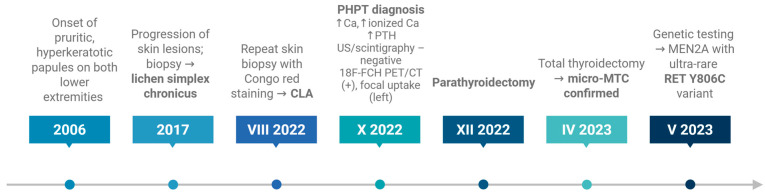
Timeline of the patient’s clinical course, illustrating the progression of cutaneous lesions, diagnostic work-up, and endocrine manifestations leading to the diagnosis of MEN2A. The figure highlights the delayed diagnosis of CLA, subsequent identification of PHPT, surgical management, and postoperative confirmation of micro-MTC, followed by genetic testing revealing an ultra-rare RET Y806C variant.

A timeline summarizing the chronological sequence of dermatological and endocrine manifestations, diagnostic procedures, and genetic testing is presented in Figure 6.

### 2.4. Follow-Up and Outcomes

**Figure 7:**
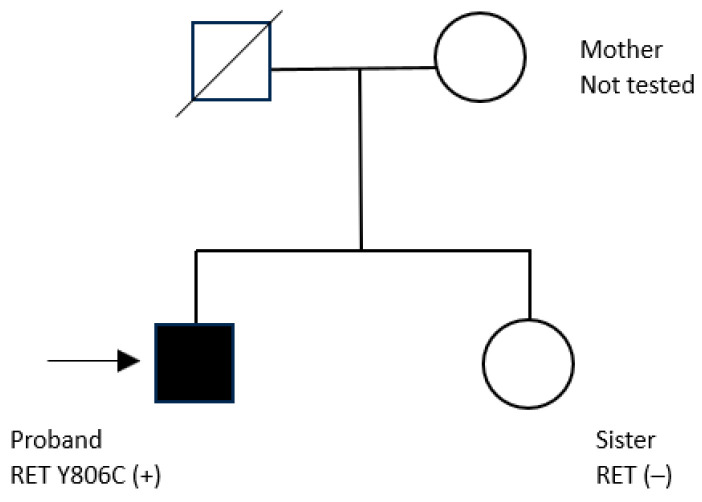
Pedigree of the proband with MEN2A (RET Y806C).

Based on the presence of MTC and PHPT, the clinical presentation was consistent with MEN2A, although no evidence of pheochromocytoma has been identified to date. The patient was therefore diagnosed with MEN2A in association with CLA. The family history is unremarkable for malignancies or hereditary disorders. The patient has no offspring. His father is deceased, and no clinical or genetic data are available. His mother, who has no history of malignancy, declined genetic testing. His sister tested negative for the RET variant and shows no clinical features consistent with MEN2A (Figure 7).

Because parental genotypes were not assessed, the possibility of familial transmission cannot be excluded, and there is no definitive evidence supporting a de novo origin. Given the limited scope of family testing, the inheritance pattern remains uncertain. Further cascade genetic testing of at-risk relatives is recommended where feasible.

## 3. Discussion

### 3.1. RET Genotype–Phenotype Correlations

The RET proto-oncogene, which encodes a tyrosine kinase receptor, plays a crucial role in the pathogenesis of MEN2A syndrome. The RET gene consists of 21 exons, with most RET alterations representing point mutations, commonly located in exons 5, 8, 10, 11, 13, 14, 15, and 16 [4]. Genetic testing for RET abnormalities is essential in suspected cases of MEN2A, with analysis typically starting with exons 10 and 11. If no pathogenic variants are found in these exons, the remaining exons are analyzed.

In MEN2A, most mutations occur at codon 634 in exon 11, involving the substitution of cysteine with arginine (C634R) [5]. Other less frequent mutations have been described at codons 631, 768, 790, 804, 844, and 891 [5]. In familial isolated form of MTC (FMTC), the most common mutated site is at codon 804 (exon 14), while other variants involve codons 768 and 790 (exon 13) [5]. MEN2B is typically associated with changes at codon 918 (exon 16). In less than 10% of MEN2B cases, variants occur at codon 883 or as compound heterozygosity involving codons 804 and 806, 904, 805, or 781 [5]. Comprehensive molecular testing is also recommended for patients with sporadic MTC, as germline RET mutations are found in approximately 6% of sporadic cases [5].

From an endocrine perspective, management of MEN2A is typically guided by genotype-based risk stratification of RET variants. However, the p.Y806C variant is not included in current risk stratification systems and remains classified as a VUS. In such cases, clinical decision-making should be guided by the patient’s phenotype. Further studies, including functional analyses and broader segregation data, are required to clarify the clinical significance of this variant and to inform future management strategies.

Our patient was diagnosed with MEN2A and CLA, a rare presentation associated with pathogenic RET variants, primarily involving codon 634. According to the literature, approximately 30% of patients with mutations affecting codon 634 have coexisting CLA [6]. Other genotype–phenotype correlations between MEN2A and CLA have been documented. RET V804M variant in exon 14 linked to MTC and CLA was reported in a 51-year-old American woman with hyperpigmented, pruritic skin lesions in the left interscapular area [7]. Additionally, a RET S891A alteration in exon 15, associated with an OSMR G513D variant, was described in members of a Chinese family with FMTC. Three family members exhibited biphasic amyloidosis (a subtype of primary cutaneous amyloidosis with concurrent lichen and macular amyloidosis). Skin lesions began with intense pruritus of the lower extremities, followed by hyperkeratotic papules that spread to the thighs, upper back, arms, and forearms, with coexisting brown macular lesions [8]. Another case of localized interscapular CLA associated with MEN2 and a RET C611Y variant was reported in a member of a Chinese family [9].

A literature search was conducted using PubMed and Scopus databases with the keywords “cutaneous lichen amyloidosis,” “MEN2A,” and “RET mutation.” Articles published in English up to 2025 were considered, and relevant case reports and case series were included. The typical locations and clinical characteristics of CLA associated with MEN2A and RET variants are summarized in Table 1.

### 3.2. Cutaneous Manifestations of MEN2A

The pathogenesis of CLA in MEN2A is not fully understood but has been linked to the condition notalgia paresthetica, characterized by chronic pruritus, paresthesia, and hyperalgesia in the interscapular region [15]. Secondary scratching may result in hyperpigmented skin changes and the development of lichen amyloidosis. CLA lesions can precede other MEN2A-associated symptoms by several years [16,17]. Some studies suggest that CLA associated with MEN2A is more common in women than men [8,9,18]. In most published cases, CLA lesions in MEN2A are localized, typically in the interscapular region [2]. Interestingly, our patient also presented with nail abnormalities, including onycholysis and onychorrhexis, which have not been previously reported in association with MEN2A-related CLA. However, these findings are nonspecific and should be interpreted with caution. Their potential relationship to amyloid deposition, chronic scratching, or an independent process remains unclear and requires further investigation.

### 3.3. Management of Cutaneous Lichen Amyloidosis

Management of CLA remains challenging due to the chronic, relapsing nature of the disease and the paucity of high-quality evidence guiding treatment decisions. Most available data derive from case reports, small case series, and a limited number of uncontrolled clinical trials. Treatment options include topical corticosteroids, calcineurin inhibitors, keratolytic agents, emollients, and phototherapy (narrowband UVB, PUVA, UVA1) [19]. Procedural approaches such as fractional CO_2_ laser, Er:YAG, Nd:YAG, non-ablative fractional lasers, and microneedling have also been reported [20]. In refractory cases, systemic therapies including retinoids, biologic agents (e.g., dupilumab, nemolizumab), and JAK inhibitors (e.g., abrocitinib) may be considered [19]. However, therapeutic responses are often variable and frequently incomplete, particularly in long-standing or extensive disease. In our patient, multiple therapeutic modalities resulted in no significant clinical improvement. Given the treatment-resistant course, biologic therapy with dupilumab is being considered as a potential option. Given the association with MEN2A, management should also include appropriate evaluation and surveillance for endocrine manifestations. Long-term prognosis of CLA is generally benign but may significantly impact quality of life due to persistent pruritus.

### 3.4. Atypical Presentation and Clinical Significance

In this case, the potentially novel aspects include the generalized distribution of CLA, its occurrence in association with a RET exon 14 (codon 806) variant, and its coexistence with PHPT, which has not been previously reported in association with this RET variant. PHPT is observed in up to 30% of patients with MEN2A and mutations in codon 634. It occurs less frequently in individuals with other RET codon mutations [21]. Moreover, in the reports published to date, there have been no documented cases of MEN2A-CLA accompanied by PHPT involving a RET variant at codons other than 634 [22].

Patients with an altered codon 634 also have a 50% risk of developing pheochromocytoma by their fifth decade of life, increasing to 90% by the eighth decade [23]. For other RET codons, the reported incidence of pheochromocytoma is significantly lower, ranging between 4% and 25% [22].

### 3.5. Limitations

In the absence of functional studies and broader segregation data, the pathogenic role of this variant cannot be definitively established, and its contribution to the observed phenotype should be interpreted with caution. As this is a single case report, these findings should be considered hypothesis-generating, and further confirmation in independent cases is required. The limited availability of family genetic data further constrains interpretation, as segregation analysis could provide important supportive evidence for clinical relevance. Therefore, additional cascade testing of at-risk relatives would be valuable in clarifying the inheritance pattern and potential pathogenicity of this variant.

### 3.6. Clinical Implications

This case highlights the importance of considering MEN2A in patients presenting with atypical or generalized CLA. While MEN2A-associated CLA is typically localized to the interscapular region and linked to RET codon 634 variants, our findings may suggest a broader phenotypic spectrum. Moreover, the initial histopathological diagnosis of lichen simplex chronicus in our patient highlights an important diagnostic pitfall in cases of long-standing pruritic papular eruptions. CLA may mimic other chronic dermatoses both clinically and histologically, particularly when amyloid deposition is subtle or not specifically assessed. In such cases, a repeat biopsy with special staining may be essential to establish the correct diagnosis. Systemic amyloidosis was not suspected, given the absence of systemic symptoms and of clinical or laboratory findings suggestive of systemic involvement; therefore, the diagnosis of primary localized cutaneous amyloidosis was considered appropriate. Dermoscopy of CLA has been reported to reveal a pattern consisting of a central white to brown hub surrounded by variably distributed brownish peripheral pigmentation. The central hub may correspond histopathologically to compact amyloid deposits within the dermal papillae, often accompanied by epidermal changes such as acanthosis and hyperkeratosis, while the peripheral pigmentation reflects melanin within amyloid deposits, basal layer hyperpigmentation, and dermal pigment incontinence [24,25]. Although such dermoscopic features have been described, their diagnostic specificity remains limited, and they should be interpreted in conjunction with clinical and histopathological findings.

Dermatologists may play a key role in early detection, particularly when pruritic, treatment-resistant, or widespread lesions precede endocrine manifestations by several years. In such cases, early genetic testing of the RET proto-oncogene may be considered to enable timely diagnosis and surveillance for associated malignancies.

## 4. Conclusions

To our knowledge, this is the first reported case of MEN2A associated with a generalized form of cutaneous lichen amyloidosis and primary hyperparathyroidism in a patient with an ultra-rare RET codon 806 variant. While the clinical and molecular findings suggest a possible association, the pathogenic role of this variant remains to be fully elucidated. This case may contribute to the expanding genotype–phenotype spectrum of MEN2A and highlights the potential role of dermatological manifestations as early indicators of underlying hereditary endocrine syndromes. Increased awareness of atypical presentations may facilitate earlier diagnosis and improve patient outcomes.

## Figures and Tables

**Table 1:** Clinical characteristics and temporal relationship of cutaneous lichen amyloidosis (CLA) in MEN2A according to RET variants. NA, not available; CLA, cutaneous lichen amyloidosis; +, positive; n, number of affected patients with CLA; MTC, medullary thyroid carcinoma; PHEO, pheochromocytoma; HPT, hyperparathyroidism.

Exon	RET Variant	CLA + (n)	Age at CLA/Pruritus Onset or Diagnosis (Years)	Location and CLA Characteristic Features	Associated Endocrine Lesions MTC/PHEO/HPT	Chronology of CLA Relative to MEN2A Manifestations	Key Reference
**10**	**C611Y**	2	40/54	Interscapular central region (T2-T6 level): brownish hyperpigmented, dry, scaly, and thickened papules with a burning sensation and episodic pruritus, worsening during periods of stress and dry weather.	MTC, PHEO	CLA preceded MEN2A diagnosis	Qi et al., 2018 [9]
**11**	**C634F**	1	57	Scapular region: brown-pigmented, hyperkeratotic papules accompanied by pruritus and occasional pain.	MTC	Scratching of the scapular region in the upper back since the age of 11 years	Fang X et al., 2022 [10]
**11**	**C634G**	3	53/54/55	On the left upper back, extending across the midline to the right side, brownish hyperpigmented, dry, and thickened papules with scabby excoriations	MTC, PHEO	CLA preceded MEN2A diagnosis 12/20/23 years correspondingly	Fang X et al., 2022 [10],
		2	NA	Localized pruritus on the back with the classical dermatological features of CLA		NA	Seri M et al., 1997 [11]
**11**	**C634R**	1	21	NA	MTC, PHEO, HPT	NA	Vieira AE et al., 2002 [12]
**11**	**C634W**	1	5	NA	MTC	NA	Lemos MC et al., 2002 [13]
**11**	**C634Y**	1	34	Interscapular region: firm, pigmented, scaly, pruritic papular lesion	MTC, PHEO	Pruritus since infancy	Gullu S et al., 2005 [14]
**14**	**V804M**	1	40	Interscapular/left scapular region: hyperpigmented lesion with excoriations, otherwise smooth-surfaced; intense episodic pruritus, worsened by stress	MTC	Pruritus for >10 years before MTC	Rothberg AE et al., 2009 [7]
**15**	**S891A**	3	28/27/31	Thighs, upper back, shoulders, arms, and forearms: lesions began as severe pruritus on lower legs, followed by lichenification and hyperpigmented brown papules; coexistence of macular lesions with rippled/reticulated appearance (biphasic amyloidosis)	MTC	CLA preceded MEN2A diagnosis	Qi XP et al., 2015 [8]
**14**	**Y806C**	1	40	Generalized hyperpigmented papules with severe pruritus; initial lesions appeared bilaterally on the lower extremities	MTC, HPT	CLA preceded MEN2A diagnosis by 16 years	This article

## Data Availability

The data presented in this study are available on request from the corresponding author.

## References

[1] Pálla S., Kuroli E., Tóth E.A., Hidvégi B., Holló P., Medvecz M. (2023). Primary Localized Cutaneous Amyloidosis in Central Europe: A Retrospective Monocentric Study on Epidemiology and Therapy. J. Clin. Med..

[2] Stanescu L.-S., Ghemigian A., Ciobica M.-L., Nistor C., Ciuche A., Radu A.-M., Sandru F., Carsote M. (2024). Thyroid Malignancy and Cutaneous Lichen Amyloidosis: Key Points Amid RET Pathogenic Variants in Medullary Thyroid Cancer/Multiple Endocrine Neoplasia Type 2 (MEN2). Int. J. Mol. Sci..

[3] Richards S., Aziz N., Bale S., Bick D., Das S., Gastier-Foster J., Grody W.W., Hegde M., Lyon E., Spector E. (2015). Standards and Guidelines for the Interpretation of Sequence Variants: A Joint Consensus Recommendation of the American College of Medical Genetics and Genomics and the Association for Molecular Pathology. Genet. Med..

[4] Miyauchi A., Futami H., Hai N., Yokozawa T., Kuma K., Aoki N., Kosugi S., Sugano K., Yamaguchi K. (1999). Two Germline Missense Mutations at Codons 804 and 806 of the RET Proto-Oncogene in the Same Allele in a Patient with Multiple Endocrine Neoplasia Type 2B without Codon 918 Mutation. Jpn. J. Cancer Res..

[5] Gopinath S., Ramaiyan V. (2024). Molecular Diagnostic Approaches in Detecting Rearranged during Transfection Oncogene Mutations in Multiple Endocrine Neoplasia Type 2. World J. Clin. Cases.

[6] Nunziata V., di Giovanni G., Lettera A.M., D’Armiento M., Mancini M. (1989). Cutaneous Lichen Amyloidosis Associated with Multiple Endocrine Neoplasia Type 2A. Henry Ford Hosp. Med. J..

[7] Rothberg A.E., Raymond V.M., Gruber S.B., Sisson J. (2009). Familial Medullary Thyroid Carcinoma Associated with Cutaneous Lichen Amyloidosis. Thyroid.

[8] Qi X.-P., Zhao J.-Q., Chen Z.-G., Cao J.-L., Du J., Liu N.-F., Li F., Sheng M., Fu E., Guo J. (2015). RET Mutation p.S891A in a Chinese Family with Familial Medullary Thyroid Carcinoma and Associated Cutaneous Amyloidosis Binding OSMR Variant p.G513D. Oncotarget.

[9] Qi X.-P., Peng J.-Z., Yang X.-W., Cao Z.-L., Yu X.-H., Fang X.-D., Zhang D.-H., Zhao J.-Q. (2018). The RET C611Y Mutation Causes MEN 2A and Associated Cutaneous Lichen Amyloidosis. Endocr. Connect..

[10] Fang X., Wang H., Dong F., Lian B., Li F., Jin H., Yu Y., Zhang N., Qi X. (2022). [Clinical and genetic analysis of seven Chinese pedigrees affected with multiple endocrine neoplasia type 2A with cutaneous lichen amyloidosis]. Zhonghua Yi Xue Yi Chuan Xue Za Zhi.

[11] Seri M., Celli I., Betsos N., Claudiani F., Camera G., Romeo G. (1997). A Cys634Gly Substitution of the *RET* Proto-Oncogene in a Family with Recurrence of Multiple Endocrine Neoplasia Type 2A and Cutaneous Lichen Amyloidosis. Clin. Genet..

[12] Vieira A.E.F., Mello M.P., Elias L.L.K., Lau I.F., Maciel L.M.Z., Moreira A.C., Castro M. (2002). Molecular and Biochemical Screening for the Diagnosis and Management of Medullary Thyroid Carcinoma in Multiple Endocrine Neoplasia Type 2A. Horm. Metab. Res..

[13] Lemos M.C., Carrilho F., Rodrigues F.J., Santos P., Carvalheiro M., Ruas M.A., Regateiro F.J. (2002). Early Onset of Medullary Thyroid Carcinoma in a Kindred with Multiple Endocrine Neoplasia Type Iia Associated with Cutaneous Lichen Amyloidosis. Endocr. Pract..

[14] Gullu S., Gursoy A., Erdogan M.F., Dizbaysak S., Erdogan G., Kamel N. (2005). Multiple Endocrine Neoplasia Type 2A/Localized Cutaneous Lichen Amyloidosis Associated with Malignant Pheochromocytoma and Ganglioneuroma. J. Endocrinol. Investig..

[15] Chabre O., Labat F., Pinel N., Berthod F., Tarel V., Bachelot I. (1992). Cutaneous Lesion Associated with Multiple Endocrine Neoplasia Type 2A: Lichen Amyloidosis or Notalgia Paresthetica?. Henry Ford Hosp. Med. J..

[16] Wells S.A., Asa S.L., Dralle H., Elisei R., Evans D.B., Gagel R.F., Lee N., Machens A., Moley J.F., Pacini F. (2015). Revised American Thyroid Association Guidelines for the Management of Medullary Thyroid Carcinoma. Thyroid.

[17] Verga U., Fugazzola L., Cambiaghi S., Pritelli C., Alessi E., Cortelazzi D., Gangi E., Beck-Peccoz P. (2003). Frequent Association between MEN 2A and Cutaneous Lichen Amyloidosis. Clin. Endocrinol..

[18] Scapineli J.O., Ceolin L., Puñales M.K., Dora J.M., Maia A.L. (2016). MEN 2A-Related Cutaneous Lichen Amyloidosis: Report of Three Kindred and Systematic Literature Review of Clinical, Biochemical and Molecular Characteristics. Fam. Cancer.

[19] Wang Q.-X., Ye Q., Zhou K.-Y., Luo S.-Y., Fang S. (2025). Systematic Review and Meta-Analysis of Treatments and Outcomes in Primary Localized Cutaneous Amyloidosis. Clin. Exp. Dermatol..

[20] Heidari N., Alaee P., Ghanavati K., Heidari A., Ghane Y., Goodarzi A. (2025). A Systematic Review of Procedural Treatment for Primary Localized Cutaneous Amyloidosis: Focus on Efficacy, Safety, Treatment Durability in Comparison and Combination. Lasers Med. Sci..

[21] Wells S.A. (2018). Advances in The Management of MEN 2- From Improved Surgical and Medical Treatment to Novel Kinase Inhibitors. Endocr. Relat. Cancer.

[22] Li S.-Y., Ding Y.-Q., Si Y.-L., Ye M.-J., Xu C.-M., Qi X.-P. (2020). 5P Strategies for Management of Multiple Endocrine Neoplasia Type 2: A Paradigm of Precision Medicine. Front. Endocrinol..

[23] Imai T., Uchino S., Okamoto T., Suzuki S., Kosugi S., Kikumori T., Sakurai A., MEN Consortium of Japan (2013). High Penetrance of Pheochromocytoma in Multiple Endocrine Neoplasia 2 Caused by Germ Line RET Codon 634 Mutation in Japanese Patients. Eur. J. Endocrinol..

[24] Sabban E.N.C., Errichetti E., Cabo H.A., Maronna E. (2022). Dermoscopy as a Supportive Tool to Differentiate Lichen Amyloidosus From Clinical Mimickers. Dermatol. Pract. Concept..

[25] Errichetti E., Stinco G. (2016). Dermoscopy in General Dermatology: A Practical Overview. Dermatol. Ther..

